# Biochanin A Ameliorates Nephropathy in High-Fat Diet/Streptozotocin-Induced Diabetic Rats: Effects on NF-kB/NLRP3 Axis, Pyroptosis, and Fibrosis

**DOI:** 10.3390/antiox12051052

**Published:** 2023-05-05

**Authors:** Chetan Ram, Shobhit Gairola, Shobhit Verma, Madhav Nilakanth Mugale, Srinivasa Reddy Bonam, Upadhyayula Suryanarayana Murty, Bidya Dhar Sahu

**Affiliations:** 1 Department of Pharmacology & Toxicology, National Institute of Pharmaceutical Education and Research (NIPER), Guwahati 781101, India; 2 Toxicology & Experimental Medicine, CSIR-Central Drug Research Institute (CDRI), Lucknow 226031, India; 3 Department of Microbiology and Immunology, University of Texas Medical Branch, Galveston, TX 77555, USA; 4 National Institute of Pharmaceutical Education and Research (NIPER), Guwahati 781101, India

**Keywords:** diabetic nephropathy, biochanin A, NLRP3 inflammasome, pyroptosis, apoptosis, TGF-β/Smad signaling

## Abstract

Nephropathy is the most prevalent microvascular disorder in diabetes mellitus. Oxidative stress and inflammatory cascade provoked by the persistent hyperglycemic milieu play integral roles in the aggravation of renal injury and fibrosis. We explored the impact of biochanin A (BCA), an isoflavonoid, on the inflammatory response, nod-like receptor protein 3 (NLRP3) inflammasome activation, oxidative stress, and fibrosis in diabetic kidneys. A high-fat-diet/streptozotocin (HFD/STZ)-induced experimental model of diabetic nephropathy (DN) was established in Sprague Dawley rats, and in vitro studies were performed in high-glucose-induced renal tubular epithelial (NRK-52E) cells. Persistent hyperglycemia in diabetic rats was manifested by perturbation of renal function, marked histological alterations, and oxidative and inflammatory renal damage. Therapeutic intervention of BCA mitigated histological changes, improved renal function and antioxidant capacity, and suppressed phosphorylation of nuclear factor-kappa B (NF-κB) and nuclear factor-kappa B inhibitor alpha (IκBα) proteins. Our in vitro data reveal excessive superoxide generation, apoptosis, and altered mitochondrial membrane potential in NRK-52E cells that were cultured in a high-glucose (HG) environment were subsided by BCA intervention. Meanwhile, the upregulated expressions of NLRP3 and its associated proteins, the pyroptosis-indicative protein gasdermin-D (GSDMD) in the kidneys, and HG-stimulated NRK-52E cells were significantly ameliorated by BCA treatment. Additionally, BCA blunted transforming growth factor (TGF)-β/Smad signaling and production of collagen I, collagen III, fibronectin, and alfa-smooth muscle actin (α-SMA) in diabetic kidneys. Our results indicate the plausible role of BCA in attenuating DN, presumably through modulation of the apoptotic cascade in renal tubular epithelial cells and the NF-κB/NLRP3 axis.

## 1. Introduction

Diabetes mellitus is growing to become a challenging metabolic disorder, and long-standing hyperglycemia is a primary predisposing factor for advancing diabetic complications [1]. Diabetic nephropathy (DN) represents a chronic long-term disorder in diabetic patients that alters the structure and function of small blood vessels. It is recognized as a prominent cause of the glomerular dysfunction, hyperfiltration, and albuminuria that eventually culminate in end-stage renal disease (ESRD) [2]. The typical histological and cellular features of diabetic kidney disease include glomerular lesions (enlarged glomerular basement membrane, mesangial hypercellularity, and extracellular matrix deposition), tubular degeneration, and interstitial matrix deposition [3]. According to a recent report, the amount of global diabetic patients (aged 18 to 99 years) is expected to escalate from 451 million in 2017 to approximately 693 million by 2045 [4]. Despite extensive research, DN remains the leading cause of morbidity and mortality among the global diabetic population and thus poses an enormous public health burden. Moreover, definitive treatment strategies against DN remain elusive. Although the precise mechanism implicated in the development of diabetic renal complications has been partially explored, a multifactorial interaction between oxidative, apoptotic, pro-inflammatory, and fibrotic pathways is suggested as crucial for the initiation and advancement of DN [1]. Several promising studies have discussed the impact of NLRP3 inflammasome and NF-κB signaling in propelling the inflammatory cascade, glomerular damage, and fibrotic changes in diabetic kidneys [5]. A multitude of mediators that are produced in diabetic conditions fuel the formation of the inflammasome assembly that consequently activates IL-1β and IL-18 in a caspase-1-mediated axis. NLRP3, a cytosolic sensor protein, interacts with a cytoplasmic protein, apoptosis-associated speck-like protein (ASC), and an execution protein, pro-caspase-1, to form a multimeric inflammasome complex. Interestingly, NF-κB is crucial in regulating the transcriptional activation of NLRP3, IL-18, and IL-1β [6]. Furthermore, cleaved caspase-1 also regulates pyroptosis, a type of programmed cell lysis featured in gasdermin-D (GSDMD)-mediated membrane pore formation. Fibrotic scarring of the glomerulus and tubulointerstitial region consequently progresses to ESRD with inexorable loss of renal function [7]. In addition, TGF-β1 has been widely studied for its distinct role in the fibrotic transformation of diabetic kidneys [8]. Inhibition of the TGF-β/Smad pathway has proven to be effective in retarding the fibrotic manifestations of DN [9,10].

Polyphenols are bioactive constituents obtained from numerous dietary plant products, including fruits, cereals, cocoa, vegetables, and legumes. Polyphenols hold strong antioxidant, immunomodulatory, and anti-inflammatory activity and contribute to human health [11]. In addition, polyphenols have been proven to be valuable in ameliorating the inflammation and fibrosis associated with diabetic nephropathy [12]. Biochanin A (BCA) (5,7-Dihydroxy-4′-methoxy-isoflavone) is a dietary isoflavonoid-type polyphenol with pronounced pharmacological benefits and is abundantly present in red clover, cabbage, soybean, chickpea, and alfalfa sprouts. It exhibits promising neuroprotective, renoprotective, cardioprotective, antidiabetic, and anti-inflammatory activities [13,14]. In our previous study, BCA was found to alleviate renal fibrosis associated with obstructive nephropathy via the inhibition of NLRP3 inflammasomes [15]. A recent study has demonstrated the therapeutic potential of orally administered BCA in streptozotocin (STZ)-induced diabetic nephropathy in rats [16]. However, the underlying mechanism of its renoprotection is poorly defined. In this study, we dissected the effect of BCA on the NF-κB (p65)/NLRP3-evoked inflammatory response, apoptosis, and expression of extracellular matrix (ECM) components in a rat model of diabetic kidney disease induced by HFD/STZ and rat kidney tubular epithelial cells (NRK-52E) cultured in a hyperglycemic environment.

## 2. Materials and Methods

### 2.1. Chemicals and Reagents

Biochanin A (purity: >98.0%, Cat. No. B4098) was obtained from TCI Chemicals Pvt. Ltd., Chennai, Tamil Nadu, India. D- (+)-Glucose (Cat. No. G8270), protease and phosphatase inhibitor cocktail (Cat. No. PPC1010), streptozotocin (Cat. No. S0130), 3-(4, 5-dimethylthiazol-2-yl)-2,5-diphenyl tetrazolium bromide (MTT), and bicinchoninic acid protein assay kit were obtained from Sigma-Aldrich, St. Louis, MO, USA. A rodent diet with 60% kcal of fat (Cat. No. D12492) was procured from Research Diets, Inc., New Brunswick, NJ, USA. Fetal bovine serum (Cat. No. A3160802), Dulbecco’s Modified Eagle’s Medium (DMEM) powder low glucose (Cat. No. 31600034), JC-1 dye (Cat. No. 65-0851-38 eBioscience™), DAPI (prolong gold antifade mountant with DAPI (Cat. No. P36931)), dihydroethidium (DHE) (Cat. No. D1168), normal goat serum (10%) (Cat. No. 50197Z), antibiotic-antimycotic (100X) (Cat. No. 15240062, Gibco, Waltham, MA, USA), secondary antibody goat anti-rabbit IgG, Alexa fluor 488 and Alexa fluor 594 (Cat. Nos. A-11034 and A-11012 respectively, Invitrogen) were obtained from Thermo Fisher Scientific, Waltham, MA, USA. Bovine serum albumin (Cat. No. MB083) and D-Mannitol (Cat. No. GRM024) were procured from HiMedia Laboratories, Thane, Maharashtra, India. All other fine chemicals were of analytical grade.

### 2.2. Cell Culture and Cytotoxicity Studies

Normal rat proximal tubular epithelial cell line (NRK-52E) was purchased from American Type Culture Collection (ATCC, Cat. No. CRL-1571, Manassas, VA, USA). Cells were grown in DMEM containing 5.56 mmol/L D-glucose with 10% fetal bovine serum and 1% antibiotic–antimycotic mix supplement in a humidified 5% CO_2_ incubator at 37 °C. A cytotoxicity assay (MTT assay) was performed to assess the optimum concentration of BCA in NRK-52E cells treated with or without high glucose (HG) (30 mmol/L), as reported in the literature [17,18]. Briefly, cells were cultured in a 96-well plate at a density of 5 × 10^3^ cells/well and incubated for 24 h. After 24 h, serum-free media containing BCA at varying concentrations (ranging from 2.5 µM to 200 µM) with or without HG were added to cells and incubated for 48 h. The percentage of the viability of cells after treatment with BCA and/or HG was calculated by considering untreated cells as 100% viable.

#### 2.2.1. Induction of Hyperglycemia and BCA Treatment

To replicate the hyperglycemic conditions in vitro, NRK-52E cells were exposed to varying concentrations of glucose and/or BCA (5, 10, and 20 µM) and divided into the following groups: (I) control, (II) high mannitol (HM), (III) HG, (IV) HG + BCA-5, (V) HG + BCA-10, and (VI) HG + BCA-20. The cells were exposed to normal (5.56 mmol/L) glucose in the control group (group I). To attain an osmotic environment equivalent to 30 mmol/L of D-glucose, cells grown in a normal glucose medium were exposed to 24.44 mmol/L of D-mannitol (HM) (group II). The cells in the HG group were stimulated with 30 mmol/L of D-glucose (group III). Additionally, cells stimulated with HG were co-treated with three different concentrations of BCA (5 μM (group IV), 10 μM (group V), and 20 μM (group VI)). The treatment, as mentioned above, was maintained for 48 h. The changes in mitochondria membrane potential, reactive oxygen species (ROS), and different proteins were assessed.

#### 2.2.2. Detection of Change in Mitochondrial Membrane Potential

The mitochondrial membrane permeability and altered potential in cells were determined by employing JC-1 staining [19]. Cells were cultivated in a 6-well plate (2 × 10^5^ cells/ well). The control and treated cells were incubated in a serum-free medium containing 2.5 μM of JC-1 dye for 30 min at room temperature. The stained cells were given a wash with phosphate buffer saline (PBS, pH 7.4), and images were captured under a fluorescence microscope (Evos imaging fluorescence microscope, Thermo Fisher Scientific, Waltham, MA, USA). The ratio of red monomers to green aggregates fluorescence was calculated.

#### 2.2.3. Determination of ROS Generation in Cells

The generation of ROS under the HG condition was assessed through DHE staining [20]. In brief, cells simultaneously treated with HG and BCA were incubated with 10 μM of DHE in serum-free media at 37 °C. After 20 min, cells were processed for fluorescence imaging under a fluorescence microscope (ThermoFisher Scientific, Waltham, MA, USA). The mean fluorescence intensity (MFI) of ROS production in the different groups was estimated through ImageJ software, NIH, Bethesda, MD, USA.

#### 2.2.4. Cell Culture Treatment for Western Blotting

Cells were maintained with complete media in a sterile culture dish (100 mm) for immunoblotting. The cells were treated with HG (30 mmol/L) to induce hyperglycemia. After that, the cells were co-incubated with 5, 10, and 20 µM BCA in serum-free media and grouped as follows: control (5.56 mmol/L D-glucose), HM (5.56 mmol/L D-glucose + 24.44 mmol/L D-mannitol), HG (30 mmol/L D-glucose), HG+ BCA-5 (30 mmol/L D-glucose and 5 µM BCA treatment), HG + BCA-10 (30 mmol/L D-glucose and 10 µM BCA treatment), and HG + BCA-20 (30 mmol/L D-glucose and 20 µM BCA treatment). After incubation for 48 h, cells were extracted and lysed using radioimmunoprecipitation (RIPA) assay buffer. The obtained cell lysate was subjected to immunoblot analysis of Bax, Bcl2, cleaved caspase-3, and GSDMD as per the procedure reported earlier [15,21].

#### 2.2.5. Immunofluorescence

The expression of proteins, including NLRP3 and caspase-1 in HG-induced NRK-52E cells, was assessed through immunofluorescence analysis [15]. Cells grown on a coverslip fixed in a 6-well plate were simultaneously incubated with BCA (5 μM, 10 μM, and 20 μM) and HG (30 mmol/L) in serum-free media. After 48 h of incubation, cells were treated with paraformaldehyde (4%), followed by permeabilization (Triton X-100 0.2%), and blocking with normal goat serum (5%). The cells were then washed and probed with primary antibodies NLRP3 (1:150 dilution, ABclonal Inc., Woburn, MA, USA) and caspase-1 (1:150 dilution, ABclonal Inc., Woburn, MA, USA), followed by washing and incubation with Alexa fluor 488 or 594 labelled anti-rabbit secondary antibody (Invitrogen, Waltham, MA, USA) at room temperature for 1 h. The nuclear stain was undertaken through DAPI stain (ThermoFisher Scientific, Waltham, MA, USA). The cells were visualized under a confocal microscope (Leica TCS SP8).

### 2.3. Animals

A total of 46 male Sprague Dawley rats (180 to 200 g body weight) were housed in the Central Animal Facility at NIPER Guwahati under a maintained temperature (22 ± 3 °C) and a relative humidity (75 ± 5%). Animals were housed under a 12 h light/dark cycle with food and water ad libitum during the study. The experimental protocol (NIPER/PC/2020/30, accessed on 9 December 2020) was endorsed by the Institutional Animal Ethics Committee (IAEC) and exhibited under the guidelines of the Committee for Control and Supervision of Experiments on Animals (CCSEA), Government of India.

### 2.4. Induction of Diabetes in Rats and Experimental Design

Rats in control groups (Group I, *n* = 6) were fed a regular diet (23% protein, 5% fats, and 53% carbohydrates), and the remaining rats (*n* = 40 numbers) were fed a high-fat diet (HFD) (60% kcal fat, Research Diets, Inc., New Brunswick, NJ, USA) for 4 weeks. On the 29th day, i.e., 1st day of the 5th week, the rats on HFD were administered a single intraperitoneal injection of STZ (30 mg/kg body weight) (Sigma-Aldrich, St. Louis, MO, USA) freshly prepared in 0.1 mol/L citrate buffer (pH = 4.5). Similarly, the control rats were intraperitoneally injected with citrate buffer. After 72 h of STZ administration, rats showing high fasting blood glucose (FBG), i.e., >200 mg/dL, were considered diabetic [22] and randomized into the following four groups. In Group II (DN, *n* = 10), rats were orally administered normal saline daily for the following 8 weeks and were provided with HFD throughout the study. In Group III (DN + BCA-10, *n* = 10), rats were orally administered BCA (10 mg/kg) daily for the following 8 weeks and were fed with HFD throughout the study. In Group IV (DN + BCA-20, *n* = 10), rats were orally administered BCA (20 mg/kg) daily for the following 8 weeks and fed with HFD throughout the study. Group V (DN + glyburide, *n* = 10, positive control) rats were administered glyburide (5 mg/kg, oral) daily for the following 8 weeks and fed with HFD throughout the study. Body weight and FBG levels were recorded once every two weeks throughout the experiment. Blood was collected at the end of the study, and serum was prepared for biochemical analysis. The kidneys and pancreas were collected and kidney weight was recorded from each animal. The samples were stored at −80 °C in a freezer for future study.

### 2.5. Assessment of Blood Urea Nitrogen and Creatinine in the Serum

The serum samples were analyzed to detect the accumulation of creatinine and blood urea nitrogen (BUN) using commercial assay kits from Accurex Biomedical Pvt. Ltd., Mumbai, Maharashtra, India. The assay was performed using a standard protocol explained in the manufacturer’s guide.

### 2.6. Assessment of Oxidative Stress Parameters in the Kidneys

The levels of reduced glutathione (GSH) and lipid peroxidation (indicated by thiobarbituric acid reactive substance (TBARS)), and the activity of glutathione s-transferase (GST) enzyme in the kidneys, were determined by employing methods described previously [21]. The superoxide dismutase (SOD) activity in the kidney homogenates of diabetic rats was determined using a commercially available kit (Sigma-Aldrich, St. Louis, MO, USA).

### 2.7. Assessment of Pro-Inflammatory Cytokines by ELISA

Kidneys were homogenized in an ice-cold PBS buffer (0.1 M, pH 7.4). Rat’s specific tumor necrosis factor (TNF)-α and interleukin (IL)-1β ELISA kits (ThermoFisher Scientific, Waltham, MA, USA) were used to quantify the concentration of TNF-α and IL-1β, respectively, in the homogenates. The instruction described by the manufacturer was followed, and the detected concentrations are illustrated in pg/mg of the protein.

### 2.8. Histopathology

The formalin-fixed kidney and pancreas tissues were paraffin-embedded and sectioned to an acceptable thickness of 4 μm. After mounting on a glass slide, sections were processed and stained with Hematoxylin and Eosin (H&E) and Masson’s Trichrome to examine microstructural changes (glomerular and tubular lesions) and collagen accumulation, respectively. The light microscopic analysis was performed by a pathologist who is unknown to the study design.

### 2.9. Immunohistochemistry (IHC)

The paraffin-embedded 4 µm sections were carefully placed on glass slides with a poly-L-lysine coat. After drying, sections were deparaffinized, processed, and heated for antigen retrieval. Subsequently, the sections were given a hydrogen peroxide (3%) treatment, blocked with goat serum, and incubated with NF-κB (p65) primary antibody (1:200 dilution) at 4 °C overnight. Afterward, the sections were washed and probed with a secondary antibody at room temperature for 60 min. The slides were stained with diaminobenzidine (DAB), followed by a counterstaining with hematoxylin for light microscopy.

### 2.10. Western Blotting

The sample preparation for immunoblotting was performed as reported earlier [15,21]. An equivalent concentration (50 μg) of protein lysate was separated on polyacrylamide gel (10%), transferred onto a nitrocellulose membrane, and probed with primary antibodies against fibrotic proteins (fibronectin, COL1A1, COL3A1, smad2/3, p-Smad2/3, TGF-β1), inflammasome-associated proteins (NLRP3, active caspase-1, active IL-1β, active IL-18, ASC, GSDMD), apoptotic proteins (cleaved caspase-3, Bax, Bcl-2), and inflammatory factors (NF-κB p65, p-NF-κB p65, IκBα, p-IκBα). The β-actin densities ensure equal loading of protein lysate in each well. All antibodies used were diluted to a ratio of 1:1000 in 1% BSA containing TBST buffer. The antibodies were obtained from ABclonal Inc., Woburn, MA, USA. The immunoreactive bands were incubated with HRP-tagged secondary antibody (Jackson Immuno Research, West Grove, PA, USA) and visualized by Super Signal West Pico Plus Chemiluminescent Substrates (Thermo Fisher Scientific, Waltham, MA, USA) on a Vilber-Fusion Western blot Chemiluminescence Imaging system. The blots were quantified by densitometry analysis using ImageJ software, NIH, Bethesda, MD, USA.

### 2.11. Statistical Analysis

GraphPad Prism version 5.0 (La Jolla, CA, USA) was employed to accomplish the statistical analysis of experimental results. All results are represented as Mean ± standard error of the mean (SEM). One-way ANOVA followed by a multiple comparison Bonferroni test was used to compare the values among different treatment groups. A *p*-value less than 0.05 reflects a statistically significant result.

## 3. Results

### 3.1. Effect of BCA on NRK-52E Cells Viability

**Figure 1:**
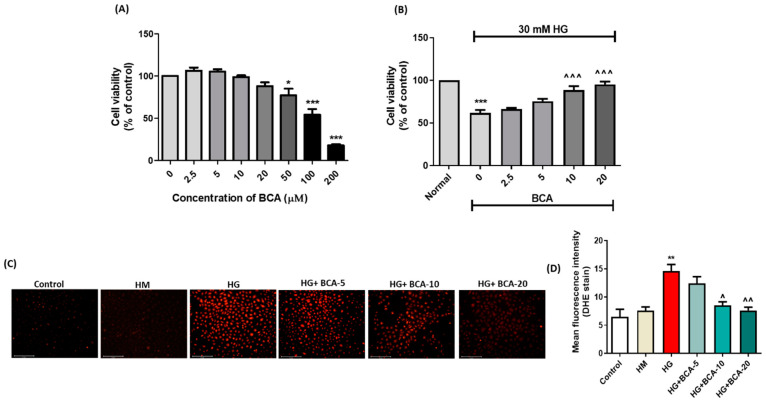
(**A**) Cell viability at different concentrations of BCA. (**B**) Effect of BCA (2.5, 5, 10, and 20 µM) on the viability of NRK-52E cells treated with HG (30 mM). (**C**) Representative images of DHE stain in NRK-52E cells. (**D**) Mean fluorescence intensity depicting the effect of BCA on HG-induced production of superoxide free radical in NRK-52E cells. The cells in the control group were grown under a low glucose medium (5.56 mmol/L D-glucose, control), and the high mannitol (HM) group (24.44 mM mannitol + 5.56 mmol/L D-glucose) was used as an osmotic control. All experimental values were expressed as mean ± SEM (*n* = 3). Where * *p* < 0.05, ** *p* < 0.01, *** *p* < 0.001 vs. control group; ˄ *p* < 0.05, ˄˄ *p* < 0.01, ˄˄˄ *p* < 0.001 vs. HG group.

In this study, we followed the MTT reduction assay principle to assess the viable cell number. The reduction of MTT into formazan crystals in living cells with metabolically active mitochondria was used as an estimate of viable cell number. Cells were treated with varying concentrations of BCA (2.5 μM to 200 μM) to determine the non-toxic concentration using an MTT reduction assay. As depicted in Figure 1A, BCA remains non-toxic at a concentration ranging from 2.5 μM to 20 μM. However, cells exposed to BCA at higher concentrations, i.e., > 50 μM, showed a significant loss in cell viability compared with the untreated cells. Based on the MTT results, we have chosen 5, 10, and 20 μM of BCA for cell-based studies.

In addition, we assessed the effect of HG (30 mM) on the viability of NRK-52E cells co-treated with BCA (2.5 μM, 5 μM, 10 μM, and 20 μM). The HG exposure showed a marked (*p* < 0.001) reduction in the viable cell population compared with the cells cultured in a normal glucose medium. BCA co-treatment at both 10 μM and 20 μM significantly (*p* < 0.001) improved the cell viability, in contrast with the HG-alone treated cells. However, BCA at 2.5 µM and 5 μM did not significantly (*p* > 0.05) improve the cell viability compared with the HG-alone treated cells (Figure 1B).

### 3.2. Effect of BCA on ROS Production in the HG-Stimulated NRK-52E Cells

Next, we evaluated the effect of BCA on HG-induced ROS generation using DHE staining (Figure 1C,D). In contrast with the untreated cells, HG-alone treated cells exhibited an intense red fluorescence (*p* < 0.01), suggesting the substantial release of ROS radicals within a hyperglycemic environment. The co-treatment with BCA markedly (*p* < 0.05 for 10 µM BCA and *p* < 0.01 for 20 µM BCA) subsided the ROS generation and intensity of DHE fluorescence compared with the HG-alone treated cells. However, BCA at 5 μM did not produce a considerable (*p* > 0.05) change in the HG-induced ROS generation.

### 3.3. Effect of BCA on HG-Induced Changes in the Mitochondrial Membrane Potential and Apoptosis Proteins in NRK-52E Cells

**Figure 2:**
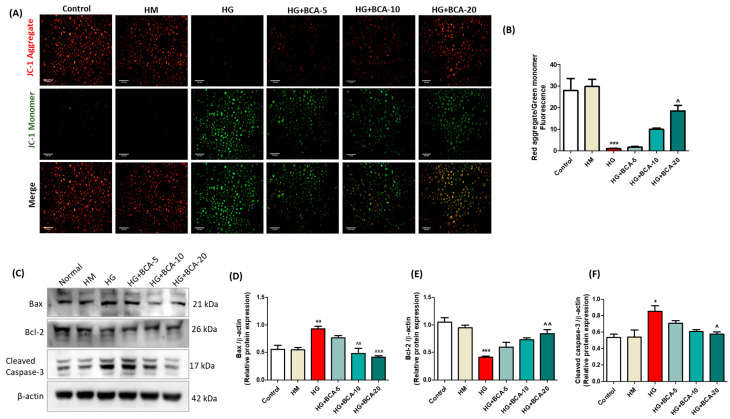
Effect of BCA on HG-induced mitochondrial dysfunction and apoptosis in NRK-52E cells. As depicted in the representative images of (**A**) JC-1 staining, a shift of the fluorescence from red (JC-1 aggregates) to green (JC-1 monomers) denotes altered membrane potential and consequent accumulation of the JC-1 monomer in mitochondria. The depolarization of the mitochondrial membrane was indicated by the (**B**) ratio of fluorescence intensity (red aggregates to green monomers). The cells in the control group were grown under a low glucose medium (5.56 mmol/L D-glucose, control), and the high mannitol (HM) group (24.44 mM mannitol + 5.56 mmol/L D-glucose) was used as an osmotic control. (**C**) Representative immunoblots and quantitative densitometric analysis of apoptotic markers (**D**) Bax, (**E**) Bcl-2, and (**F**) cleaved caspase-3 relative to β-actin. Three independent sets of experiments were performed, and the values are represented as mean ± SEM. Where * *p* < 0.05, ** *p* < 0.01, *** *p* < 0.001 vs. control group; ˄ *p* < 0.05, ˄˄ *p* <0.01, ˄˄˄ *p* < 0.001 vs. HG group.

The mitochondrial dysfunction in hyperglycemic conditions was evaluated using JC-1 fluorescent dye. As represented in Figure 2A,B, the untreated control cells stained with JC-1 dye exhibited intense red fluorescence. In contrast, the HG-alone treated cells showed a fluorescent emission shift from red to green, resulting in a marked decrease (*p* < 0.001) in the red aggregate to green monomer fluorescence ratio. The cells co-treated with BCA remarkably (BCA 20 µM: *p* < 0.05) restored the red-to-green fluorescence ratio compared with the HG-alone treated cells indicating the stabilization of mitochondrial membrane potential.

Renal tubular epithelial cell apoptosis is a prominent feature of kidney damage during diabetes. Therefore, we assessed the pro-apoptotic and anti-apoptotic proteins in NRK-52E cells exposed to hyperglycemic conditions (Figure 2C–F). We found a significant upregulation of Bax (*p* < 0.01) and cleaved caspase-3 (*p* < 0.05), in the HG-alone treated cells in contrast with the control cells. Meanwhile, the expression of Bcl-2 was reduced (*p* < 0.001) in the HG-alone-induced cells. Co-treatment with BCA 20 µM restored the Bcl-2 (*p* < 0.01) expression and decreased Bax (*p* < 0.001) and cleaved caspase-3 (*p* < 0.05) proteins compared to the HG-alone treated cells. However, the Bcl-2 and cleaved caspase-3 proteins were statistically unchanged (*p* > 0.05) in BCA 5 and 10 µM treated cells compared to the HG-alone cells.

### 3.4. Effect of BCA on HG-Induced NLRP3, Caspase-1, and Pyroptosis in NRK-52E Cells

**Figure 3:**
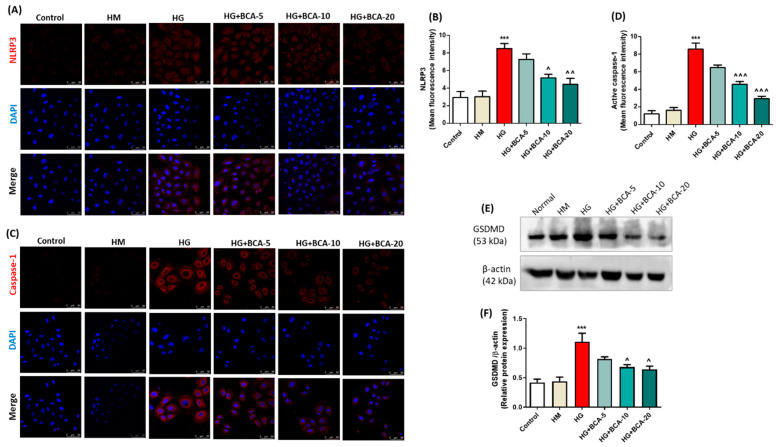
Effect of BCA on NLRP3, active caspase-1, and GSDMD expression in NRK-52E cells. The cells in the control group were grown under a low glucose medium (5.56 mmol/L D-glucose, control), and the high mannitol (HM) group (24.44 mmol/L mannitol + 5.56 mmol/L D-glucose) was used as an osmotic control. Immunofluorescence analysis was performed to detect the expression of (**A**) NLRP3 and (**C**) active caspase-1 in NRK-52E cells cultured in high glucose conditions. Immunofluorescence images were captured at a scale bar of 50 µm. The bar graph represents the mean fluorescence intensity of (**B**) NLRP3 and (**D**) active caspase-1. (**E**) Representative immunoblots and (**F**) relative protein expression of GSDMD in HG-induced NRK-52E cells. β-actin was employed as a loading control. Results are expressed as mean ± SEM of at least three independent experiments. Where *** *p* < 0.001 vs. control group; ˄ *p* < 0.05, ˄˄ *p* < 0.01, ˄˄˄ *p* < 0.001 vs. HG group.

To establish the contribution of inflammasome signaling in the progression of nephropathy in hyperglycemic conditions, inflammasome activation markers and GSDMD, an indicator of pyroptosis was assessed in the HG-induced NRK-52E cells (Figure 3). We performed immunofluorescence analysis and found elevated expression of NLRP3 (*p* < 0.001) (Figure 3A,B) and active caspase-1 (*p* < 0.001) (Figure 3C,D) in cells stimulated with HG-alone compared to the unstimulated control cells. However, the mean fluorescent intensities of NLRP3 (BCA 10 µM: *p* < 0.05; BCA 20 µM: *p* < 0.01) and active caspase-1 (*p* < 0.001 at both 10 and 20 µM BCA) were found to be markedly reduced after treatment with BCA. Additionally, no significant change was observed in NLRP3 and active caspase-1 protein in the BCA 5 μM-treated group compared to the HG-alone treated cells. Next, we assessed the expression of the pyroptosis marker protein, GSDMD, through immunoblotting (Figure 3E,F). HG exposure significantly (*p* < 0.001) induced the expression of GSDMD, whereas co-treatment with BCA 10 and 20 μM markedly (*p* < 0.05) prevented the rise in GSDMD levels in NRK-52E cells compared with the HG-alone treated cells.

### 3.5. Effect of BCA Treatment on Body Weight Changes, FBG, and Serum Kidney Injury Biomarkers in Diabetic Rats

**Figure 4:**
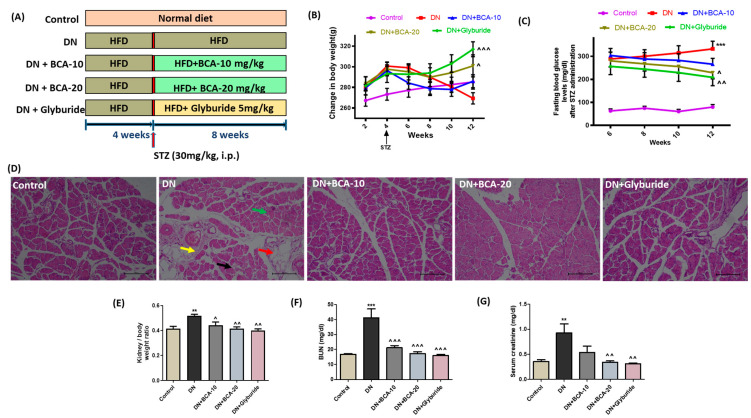
(**A**) Study design. Effect of BCA on (**B**) change in body weight and (**C**) fasting blood glucose (FBG) level. (**D**) Representative images of H&E staining (20×) of the pancreas (*n* = 3). DN group shows the diminution of pancreatic β-cell acini, degeneration of parenchyma (black arrow), fibrinous degeneration (yellow arrow), inflammatory edema (green arrow), and fatty degeneration (red arrow). DN + BCA-10 group shows mild protection with inflammatory edema. DN + BCA-20 group and DN+ glyburide-treated groups show marked protection with minimal changes compared with the DN group. Effect of BCA on (**E**) percent change in body weight to kidney weight ratio, (**F**) blood urea nitrogen (BUN), and (**G**) serum creatinine level in diabetic rats. Data are presented as mean ± SEM (*n* = 6 to 8 animals per group). Where ** *p* < 0.01, *** *p* < 0.001 vs. control group; ˄ *p* < 0.05, ˄˄ *p* < 0.01, ˄˄˄ *p* < 0.001 vs. DN group.

The detailed experimental design is shown in Figure 4A. We observed a marked decline in the body weight of HFD-fed rats after STZ injection. The end-day body weight of the DN group animals was considerably decreased compared with those of the control group. The treatment with BCA (20 mg/kg) recovered the body weight of diabetic rats (Figure 4B). Next, we estimated the FBG level of all experimental groups on the 6th, 8th, 10th, and 12th weeks of the study to determine the glycemic index (Figure 4C). In contrast with the control group, the DN group exhibited constantly elevated (*p* < 0.001) FBG levels indicating altered glucose homeostasis. Treatment with BCA 20 mg/kg markedly reduced (*p* < 0.05) the FBG, whereas FBG levels remained unchanged after BCA 10 mg/kg administration. On the other hand, standard treatment (glyburide) substantially decreased the FBG level (*p* < 0.01) and prevented weight loss (*p* < 0.001) compared with the DN group. Further, to support the glucose-lowering effect of BCA, we evaluated the histological changes in the pancreas. As expected, we observed a diminution of pancreatic β-cell acini, degeneration of parenchyma, fibrinous degeneration, inflammatory edema, and fatty degeneration in the DN group. In BCA (20 mg/kg) and glyburide-treated groups, we observed marked protection with minimal changes compared with the DN group (Figure 4D).

In addition, we found a higher kidney weight to body weight ratio in the rats of the DN group than in the control group animals (Figure 4E). The serum kidney injury biomarkers, BUN (Figure 4F) and creatinine (Figure 4G) were also significantly (*p* < 0.001 for BUN and *p* < 0.01 for creatinine) elevated in the DN group. The oral intervention of BCA markedly reduced the kidney weight to body weight ratio (*p* < 0.05 at BCA 10 mg/kg; *p* < 0.01 at BCA 20 mg/kg), BUN (*p* < 0.001 at both BCA 10 and 20 mg/kg), and creatinine (*p* < 0.01 at BCA 20 mg/kg). No significant change was noted in the serum creatinine concentration in the BCA 10 mg/kg group. The glyburide treatment also markedly attenuated the increase in kidney weight to body weight ratio, BUN, and creatinine levels compared with the DN group.

### 3.6. Effect of BCA on Kidney Histopathological Changes and Antioxidant Status

**Figure 5:**
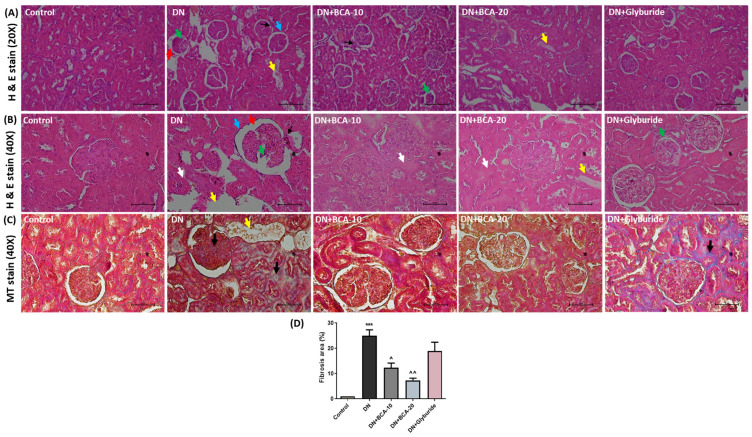
Effect of BCA on histological changes in diabetic kidneys. Kidney sections from each experimental group (*n* = 3) were stained with H&E ((Panel **A** (20×) and Panel **B** (40×)) and Masson’s trichrome (Panel **C** (40×)). (**A**) Representative images of H&E staining depict normal glomerular and tubular architecture in the control group. At the same time, the DN group exhibits enhanced glomerular basement membrane thickness (black arrow), increased glomerular volume (blue arrow), widening of subcapsular Bowman’s space (red arrow), shrunken glomeruli (green arrow), massive degeneration of tubular epithelium (yellow arrow), and renal tubular epithelial cells edema (white arrow) in the cortex region. Both DN + BCA-10 and DN + BCA-20 groups demonstrated occasional tubular epithelial edema, reduced glomerular basement membrane thickness, minimum widening of subcapsular Bowman’s space, and few shrunken glomeruli. The sections from the glyburide-treated group display minimal glomerular and tubular alterations. (Panel **C**) Fibrosis in diabetic kidneys was examined through Masson’s trichrome (MT) staining. Representative photomicrographs of control kidney sections displayed no evident collagen accumulation. The DN group witnessed massive collagen deposition (black arrowhead) indicated by a large collagen-positive area in the cortex region of the kidneys. However, the DN + BCA-10 group showed a reduced collagen synthesis with moderate fibrosis area. The sections from the DN + BCA-20 group exhibit minimum fibrotic changes and significantly low collagen buildup. In the DN + glyburide group, we did not find a significant decrease in collagen accumulation compared with the DN group. Graphical illustration of (**D**) percent fibrosis area (*n* = 3). Where *** *p* < 0.001 vs. control group; ˄ *p* < 0.05 and ˄˄ *p* < 0.01 vs. DN group.

To assess the glomerular and tubular lesions in diabetic kidneys, the kidney histology was examined through H&E staining and light microscopy (Figure 5, Panel A and Panel B). The sections from the control group showed intact glomerulus and normal renal tubules. The sections from DN groups showed an increased glomerular basement membrane thickness (GBM), degeneration of both proximal and distal renal tubules, widening of subcapsular bowman’s space, and increased glomerular volume. The DN + BCA-10mg group showed marked improvement in kidney histology, reduced vacuolar degeneration of tubules, GBM thickness, and glomerular volume compared with the DN group. The DN + BCA-20mg group showed intact GBM thickness and glomerular volume and occasional tubular degeneration compared with the DN group. The kidney sections from glyburide-treated rats demonstrated a marked reversal of histological abnormalities with minimal glomeruli and renal tubule damage. Furthermore, Masson’s trichrome staining provided the extent of collagen synthesis and accumulation in the kidneys (Figure 5, Panel C). The control group illustrated no evident fibrotic changes and kidney collagen accumulation. The section from the DN group exhibited abnormal synthesis and collagen deposition in the kidneys. The sections from the DN + BCA-10mg group showed mild synthesis and accumulation of collagen compared with the DN group. However, the sections from the DN + BCA-20mg group showed predominant intact histology without evidence of collagen accumulation. We did not observe a significant reduction of collagen deposition in the glyburide administration group of rats.

**Figure 6:**
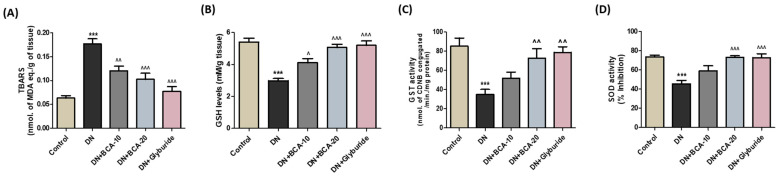
Effect of BCA on antioxidants and lipid peroxidation levels in diabetic kidneys. (**A**) Thiobarbituric acid reactive substance (TBARS) level, (**B**) reduced glutathione (GSH) level, (**C**) glutathione s-transferase (GST) activity, and (**D**) superoxide dismutase (SOD) activity. The results are expressed as mean ± SEM (*n* = 6 to 8 animals per group). Where *** *p* < 0.001 vs. control group; ˄ *p* < 0.05, ˄˄ *p* < 0.01, ˄˄˄ *p* < 0.001 vs. DN group.

Next, we assessed the lipid peroxidation and antioxidant status in the kidneys. In contrast with the control animals, the DN group exhibited substantially elevated (*p* < 0.001) levels of TBARS, a lipid peroxidation marker in the kidneys (Figure 6A). In addition, we observed a marked (*p* < 0.001) depletion of renal antioxidants, namely, GSH (Figure 6B), GST (Figure 6C), and SOD (Figure 6D), in the DN group of rats. The administration of BCA markedly subsided the augmented TBARS level compared with the DN group. Furthermore, renal GSH levels (BCA 10 mg/kg: *p* < 0.05; BCA 20 mg/kg: *p* < 0.001), GST (BCA 20mg/kg: *p* < 0.01), and SOD activity (BCA 20 mg/kg: *p* < 0.001) were markedly restored in the BCA treatment group. However, BCA 10 mg/kg treatment remains ineffective in recovering the renal antioxidants (GST and SOD) in contrast with the DN group. Moreover, the treatment with glyburide also restored renal antioxidant status as indicated by an improved GSH level, enhanced activity of GST and SOD, and reduced levels of TBARS, in contrast with the DN group.

### 3.7. Effect of BCA on ECM Components and TGF-β/Smad Axis in Diabetic Kidneys

**Figure 7:**
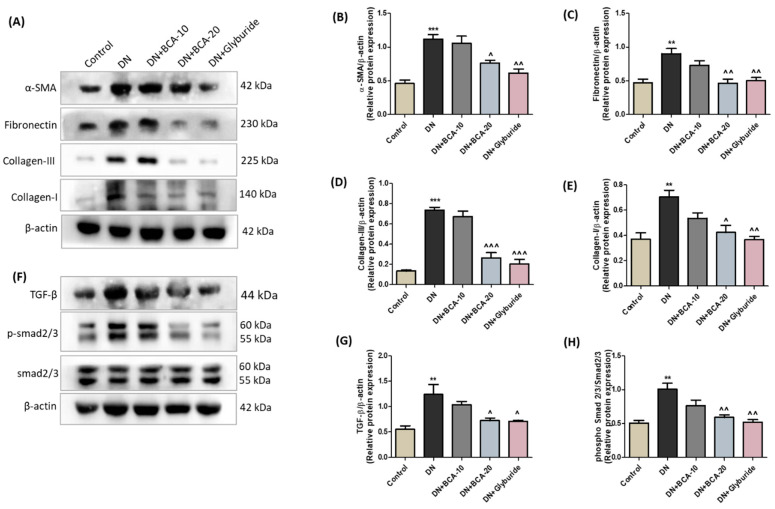
Effect of BCA on TGF-β/Smad signaling and expression of fibrotic matrix proteins. (**A**) Representative Western blots and bar diagram showing expression of (**B**) α-SMA, (**C**) fibronectin, (**D**) collagen III, and (**E**) collagen I proteins. (**F**) Representative Western blots and bar diagram showing expression of (**G**) TGF-β and (**H**) phospho-Smad2/3/Smad2/3 proteins. Data are represented as mean ± SEM (*n* = 3). Where ** *p* < 0.01, *** *p* < 0.001 vs. control group; ˄ *p* < 0.05, ˄˄ *p* < 0.01, ˄˄˄ *p* < 0.001 vs. DN group.

We further assessed the effect of BCA on diabetes-induced renal fibrogenesis by evaluating various ECM proteins using immunoblot analysis (Figure 7). We observed an elevated amount of fibrotic (*p* < 0.001 for α-SMA and collagen III; *p* < 0.01 for fibronectin and collagen I) proteins in the kidneys of the DN group. Administration of BCA 20 mg/kg and glyburide (5 mg/kg) substantially alleviated the abnormal production of these fibrotic proteins compared with the DN group. In addition, our results suggest a marked perturbation of the renal TGF-β/Smad signaling axis in the DN group with a sharp rise in TGF-β (*p* < 0.01) and p-Smad 2/3 (*p* < 0.01) protein expression in contrast with the non-diabetic control rats. The oral intervention of BCA 20 mg/kg significantly reduced the TGF-β (*p* < 0.05) and p-Smad 2/3 (*p* < 0.01) proteins. Moreover, the glyburide-treated rats also demonstrated a reduced level of TGF-β and p-Smad2/3 compared with the DN group. However, we did not find any effect on the expression of TGF-β, p-Smad2/3, and other ECM components in the BCA 10 mg/kg treatment group.

### 3.8. Effect of BCA on Renal NLRP3/Caspase-1 Signaling Axis in Diabetic Kidneys

**Figure 8:**
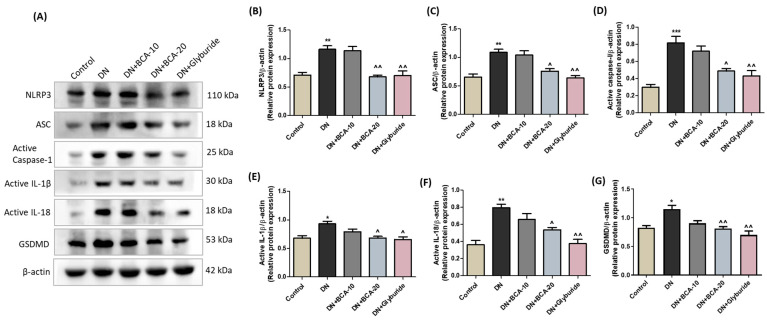
Effect of BCA on activation of NLRP3 signaling axis in diabetic kidneys. (**A**) Western blot analysis assessed the expression of NLRP3 and downstream components. Densitometric analysis of (**B**) NLRP3, (**C**) ASC, (**D**) active caspase-1, (**E**) active IL-1β, (**F**) active IL-18, and (**G**) GSDMD. β-actin was employed as a loading control. Experimental values are presented as mean ± SEM (*n* = 3). Where * *p* < 0.05, ** *p* < 0.01, *** *p* < 0.001 vs. control group; ˄ *p* < 0.05, ˄˄ *p* < 0.01 vs. DN group.

Further, we studied the NLRP3 inflammasome signaling in diabetic kidneys. As depicted in Figure 8, Western blot analysis confirmed the pronounced elevation of active IL-1β (*p* < 0.05), active IL-18 (*p* < 0.01), active caspase-1 (*p* < 0.001), NLRP3 (*p* < 0.01), ASC (*p* < 0.01), and GSDMD (*p* < 0.05) proteins in the DN group. BCA intervention at 20 mg/kg significantly reduced the inflammasome proteins (*p* < 0.01 for NLRP3 and GSDMD; *p* < 0.05 for ASC, active caspase-1, active IL-1 β, and active IL-18) compared with the DN group. Moreover, glyburide treatment also demonstrated a significant inhibition of NLRP3, ASC, active caspase-1, IL-1β, IL-18, and GSDMD proteins. However, NLRP3 and the associated components of inflammasome remained statistically unchanged in the BCA 10 mg/kg treatment group. These results collectively indicate that BCA at 20 mg/kg modifies the NLRP3/caspase-1 signaling cascade and alleviates inflammation in diabetic kidneys.

### 3.9. Effect of BCA on NF-kB (p65) and Pro-Inflammatory Cytokines in Diabetic Kidneys

**Figure 9:**
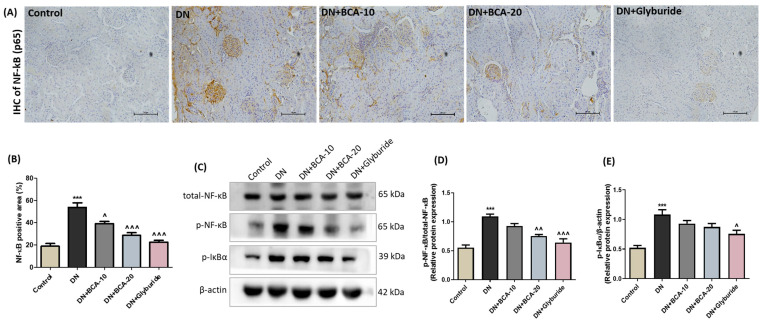
Effect of BCA on NF-κB-mediated renal inflammation in diabetic kidneys. (**A**) Representative images of kidney sections probed with primary antibodies against NF-κB (p65). The photomicrographs of the control group depict minimal NF-κB (p65) expression. In the DN group, the glomeruli in the cortex region show significant expression of NF-κB (p65). BCA-treated groups (DN + BCA-10 and DN + BCA-20) revealed a markedly reduced NF-κB (p65) positive area, and the standard treatment group (DN + glyburide) exhibited marginal NF-κB (p65) expression. (**B**) Quantification of NF-κB (p65) positive area. (**C**) Representative Western blots and quantitative analysis of (**D**) p-NF-κB(p65) relative to total-NF-κB (p65) and (**E**) p-IκBα relative to β-actin. Results are presented as mean ± SEM (*n* = 3). Where *** *p* < 0.001 vs. control group; ˄ *p* < 0.05, ˄˄ *p* < 0.01, ˄˄˄ *p* < 0.001 vs. DN group.

The effect of BCA in diabetes-induced inflammatory response was evaluated by estimating the proteins of the NF-κB signaling axis and other pro-inflammatory cytokines in the kidneys. IHC study revealed an increased NF-κB (p65) positive area in the renal cortical region of the diabetic kidneys compared with the control group, while BCA treatment significantly reduced the cortical expression of NF-κB (p65). Furthermore, the immune blot analysis also confirmed the higher levels of p-NF-κB (p65) and p-IκBα in the kidneys of the DN group. BCA administration at 20 mg/kg inhibited the phosphorylation of NF-κB (p65) (*p* < 0.01). The standard glyburide treatment also decreased (*p* < 0.001) the NF-κB (p65) positive area and the phosphorylation of NF-κB and IκBα (Figure 9). However, these inflammatory markers remain statistically unchanged in the BCA 10 mg/kg treatment group.

**Figure 10:**
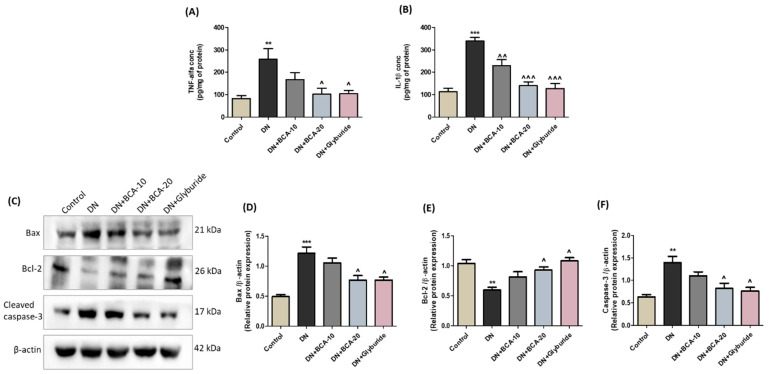
Effect of BCA on the production of pro-inflammatory cytokines and apoptotic proteins in diabetic kidneys. The concentration of (**A**) TNF-α and (**B**) IL-1β estimated through ELISA (*n* = 5 to 6 animals per group). Activation of the apoptotic cascade in kidneys indicated by the (**C**) immunoblots and respective bar diagrams of (**D**) Bax, (**E**) Bcl-2, and (**F**) cleaved caspase-3 (*n* = 3). β-actin was employed as a control protein. Results are presented as mean ± SEM. Where ** *p* < 0.01, *** *p* < 0.001 vs. control group; ˄ *p* < 0.05, ˄˄ *p* < 0.01, ˄˄˄ *p* < 0.001 vs. DN group.

Next, we explored the impact of BCA intervention on the inflammatory cascade in diabetic kidneys. We found an intense production of TNF-α (*p* < 0.01) (Figure 10A) and IL-1β (*p* < 0.001) (Figure 10B) in the kidney lysate of the DN group compared with the control group. The oral intervention of BCA significantly decreased the levels of TNF-α (BCA 20 mg/kg: *p* < 0.05) and IL-1β (BCA 10 mg/kg: *p* < 0.01; BCA 20 mg/kg: *p* < 0.001). Glyburide administration also blunted the production of both TNF-α and IL-1β compared with the DN group.

### 3.10. Effect of BCA on Apoptosis-Associated Proteins in Diabetic Kidneys

We determined the expression of Bax, Bcl-2, and cleaved caspase-3 to assess hyperglycemia-associated apoptotic cell death in the kidneys (Figure 10C–F). In agreement with the in vitro data, renal damage in the DN group of rats was indicated by a decline in the Bcl-2 protein expression (*p* < 0.01) and an augmented Bax (*p* < 0.001) and cleaved caspase-3 (*p* < 0.01) protein expression compared with the control group. However, the oral intervention of both BCA (20 mg/kg) and glyburide markedly (*p* < 0.05) restored the level of Bcl-2 proteins and ameliorated the expression of pro-apoptotic Bax and cleaved caspase-3 proteins. On the other hand, BCA 10 mg/kg treatment was ineffective in inhibiting the apoptotic cascade in the kidneys of diabetic rats.

## 4. Discussion

Nephropathy is considered one of the most prevalent microvascular disorders in diabetic patients. Lately, DN has begun to appear as a major risk factor for ESRD, a devastating condition with significant loss of renal function and few treatment alternatives, such as dialysis or renal transplant. In clinical settings, the therapeutic benefits of current treatments for DN are still limited, and alternative therapies, including phytomedicine, might offer additional benefits to the patients. In the present work, an experimental model of DN was established in rats through STZ injection combined with HFD for 12 weeks. Furthermore, the diabetic rats were orally treated with BCA for 8 consecutive weeks (i.e., 4th week to 12th week). The effect of BCA on oxidative stress, activation of the NF-κB/NLRP3 signaling axis, and interstitial fibrosis was assessed in diabetic kidneys and renal tubular epithelial cells cultured in an HG environment. Consistent with the earlier reports, the induction of diabetes in HFD-fed/STZ-induced rats was manifested by persistent hyperglycemia and weight loss in the DN group [23]. Further, the functional deficit in the kidneys of diabetic rats was marked by elevated BUN and serum creatinine levels, reflecting their altered glomerular clearance. However, the oral intervention of BCA at 20 mg/kg significantly inhibited the weight loss, restored the blood glucose to normal, and improved the glomerular filtration of BUN and creatinine compared with the DN group. The increased kidney weight to body weight ratio and abnormalities in renal histoarchitecture, including GBM thickening, were also mitigated after BCA treatment. Glyburide-treated rats demonstrated a moderate change in body weight and normal FBG levels compared with the DN group. Moreover, the renal histological changes and glomerular changes were normalized after glyburide treatment.

Persistent hyperglycemia coupled with a diabetic milieu is known to perturb the cellular redox balance leading to the unchecked production of ROS, a condition denoted as oxidative stress. Accumulating evidence substantiates the imperative role of oxidative stress in diverse pathological states, including DN [24]. Moreover, oxidative stress is recognized as a crucial factor that aggravates renal inflammation, apoptosis, and fibrosis in diabetic kidneys [25]. Herein, we assessed the impact of BCA on renal oxidative damage, HG-induced ROS generation, and mitochondrial dysfunction in NRK-52E cells. Although excessive ROS generation in the kidneys of diabetic rats is accompanied by lipid peroxidation and exhaustion of the cellular antioxidant reservoir, BCA treatment demonstrated a marked reversal of the renal antioxidant capacity, indicated by improved levels of GSH, GST, and SOD. Furthermore, our in vitro results indicate augmented superoxide generation in NRK-52E cells in response to HG. Co-treatment with BCA (10 and 20 µM) significantly reduced the production of superoxide radicals. Oxidative stress is postulated to induce an apoptotic program in renal tubular epithelial cells, resulting in tubular damage and progression of DN. Indeed, pronounced expression of apoptotic markers in diabetic kidneys significantly correlates with high oxidative stress and declining renal function [26]. Mitochondrial dysfunction is presumably a prominent feature of early apoptotic cell death. In essence, alteration in mitochondrial membrane potential in a hyperglycemic state encourages the release of pro-apoptotic factors [27]. In line with the previous studies [28,29], HG exposure was manifested by a marked disruption of mitochondrial membrane potential, loss of Bcl-2 expression, and augmentation of the apoptotic markers Bax and cleaved caspase-3 in NRK-52E cells. Similarly, kidney damage in diabetic rats was accompanied by a profound expression of Bax and cleaved caspase-3. BCA treatment improved Bcl-2 level and attenuated Bax and cleaved caspase-3 expression in diabetic kidneys and HG-induced NRK-52E cells. These findings reveal the prominent role of oxidative stress and apoptosis associated with the hyperglycemic milieu in the progression of diabetic kidney disease. Moreover, BCA was found to indirectly mitigate renal tubular cell apoptosis and mitochondrial dysfunction through its potent antioxidant activity.

Renal inflammation provoked by a hyperglycemic milieu is distinguished as a determinant factor for diabetic kidney disease. A sequence of interlinked events, including the local synthesis of chemotactic factors, an intense influx of immune cells, and subsequent release of inflammatory cytokines, encourage the inflammatory cascade in diabetic kidneys [23]. Numerous studies have reported an excessive renal influx of circulating immune cells in diabetic animals and overexpression of certain pro-inflammatory cytokines in the kidneys [30,31]. Notably, the transcriptional regulation of these inflammatory factors is governed by the NF-kB signaling axis. In the cytosol, IkB confronts the nuclear translocation of NF-kB and renders it in a latent state. Moreover, diabetes-associated hyperglycemia and oxidative stress in kidneys trigger the activation cascade of NF-kB, resulting in the transcription of mediators implicated in renal inflammation [32]. Accumulating evidence has also revealed that BCA treatment alleviates inflammation associated with metabolic, pulmonary, and neuronal disorders through modulation of the NF-kB signaling axis [14]. Our experimental data have substantiated the profound activation of NF-kB (p65)-mediated inflammation in kidneys, as indicated by the overexpression of p-IκB and p-NF-κB (p65) proteins in the DN group compared with the control group. However, BCA administration resolved renal inflammation by abolishing the phosphorylation of NF-κB (p65). Furthermore, the aberrant inflammatory damage aggravated by TNF-α and IL-1β in kidneys was markedly decreased after BCA treatment. As expected, the glyburide intervention also significantly mitigated renal inflammatory changes. For instance, the kidneys of glyburide-treated rats demonstrated decreased expression of p-IκB and p-NF-κB (p65) proteins and a marginal level of TNF-α and IL-1β. These findings highlight the distinct role of the NF-κB (p65) signaling axis in fostering renal inflammation during diabetes conditions. Furthermore, our results indicate the pronounced inhibition of NF-κB (p65) signaling and inflammatory outcomes after BCA administration to diabetic rats.

In recent years, the pathological role of NLRP3 inflammasome in renal disorders has drawn substantial attention [33]. Several studies have proven that the expression of NLRP3 inflammasome components is closely linked with renal inflammation and fibrosis [34]. In line with these studies, our previous work has also revealed the perturbed appearance of NLRP3, IL-1β, and IL-18 in the kidneys of the disease group [15]. The interaction between NF-κB and the NLRP3 inflammasome is complex and poorly understood. However, studies have recognized that NF-κB is crucial for priming NLRP3 inflammasome [35]. Upon activation, NLRP3 inflammasome triggers the caspase-1-dependent maturation of IL-18 and IL-1β. Additionally, the maturation of caspase-1 has been shown to regulate a cell death program associated with GSDMD-mediated cell lysis and subsequent expansion of the inflammatory milieu [36]. Therefore, targeting the NLRP3 inflammasome might be an effective strategy for regulating inflammation and fibrosis in diabetic kidney disease. Consistent with earlier reports, the progression of kidney disease in diabetic rats was associated with the perturbation of the NLRP3 inflammasome signaling axis. For instance, increased expression of NLRP3, ASC, and active caspase-1 was witnessed in the DN group. However, BCA 20 mg/kg intervention markedly ameliorated the protein level of inflammasome components along with reducing the expression of IL-18 and IL-1β. Additionally, GSDMD-mediated pyroptotic cell death in diabetic kidneys was also attenuated by BCA. Our in vitro data also indicate the inhibition of NLRP3 inflammasome signaling and pyroptosis in HG-induced NRK-52E cells exposed to BCA at 10 and 20 µM concentrations. Collectively, our results substantiate the crucial role of the NLRP3 inflammasome in DN and the inhibitory part of BCA on its activation.

Renal fibrosis remains a challenging clinical manifestation of DN. Although TGF-β is known to promote tissue repair as a physiological response, high glucose could induce the pathological activation of canonical TGF-β signaling, resulting in exorbitant matrix deposition and kidney fibrosis [8]. The canonical arm of the TGF-β pathway functions through adaptor proteins (Smad2, Smad3, and Smad4). TGF-β initiates the phosphorylation cascade of these proteins, forming a trimeric complex that migrates to the nucleus and transcribes various fibrotic genes [37]. Elevated expression of TGF-β and downstream signaling components have been reported in patients and murine DN models [8,38]. In agreement with earlier literature [39,40], persistent hyperglycemia stimulated canonical TGF-β signaling, as evidenced by increased TGF-β and phosphorylated Smad2/3 in the kidneys of the DN group. Furthermore, the progression of kidney fibrosis in diabetic rats was manifested by substantial production of collagen, fibronectin, and α-SMA. Interestingly, the increased TGF-β, the phosphorylated Smad 2/3 expression, and fibrotic changes in the kidneys were significantly attenuated by BCA 20 mg/kg oral treatment.

The current study explored the beneficial effect of BCA against HFD/STZ-induced diabetic kidney disease in rats. However, our study has several limitations that need to be addressed. BCA is a poorly soluble flavonoid and studies have documented that the bioavailability of BCA is poor (< 4%) [41,42]. We could not provide bioavailability and biodistribution study data of the BCA that reaches the kidneys after oral administration, this needs further investigation in order to facilitate understanding of the direct effect of BCA on the kidneys. Another limitation is that we could not measure the food and water intake of rats during the study. However, the rats were given food and water *ad libitum*. The findings of our study strongly indicate the renoprotective effect of BCA via inhibition of the NF-κB/NLRP3 axis. However, it will be interesting to examine the detailed mechanism of BCA by implying the loss of function (siRNA/shRNA) or gain of function studies against different molecular targets.

## 5. Conclusions

The outcomes of the present study reveal the prominent role of the NLRP3 inflammasome and tubular epithelial cell apoptosis in DN. Persistent hyperglycemia, coupled with diabetes, exacerbates renal dysfunction and severe histological damage in the DN group. Oral administration of BCA alleviated renal injury markers (BUN and serum creatinine) and recovered antioxidant levels and histological changes. Our in vitro experiments indicate that BCA attenuated apoptotic cell death and the NLRP3 inflammasome in NRK-52E cells cultured in HG. Moreover, BCA improved altered mitochondrial membrane potential and suppressed ROS production in NRK-52E cells. In line with the in vitro results, the oral intervention of BCA in diabetic rats downregulated pro-apoptotic proteins, inflammasome components, and renal inflammation. Furthermore, BCA blunted the TGF-β/Smad pathway and the formation of ECM components in the kidneys of diabetic rats. In conclusion, our results strongly indicate the protective effect of oral BCA against diabetic kidney diseases.

## Data Availability

Data is contained within the article.
